# A biomarker approach to syndrome-based treatment of severe childhood illness in malaria-endemic areas

**DOI:** 10.1186/s12936-018-2533-9

**Published:** 2018-10-22

**Authors:** Hans Ackerman, Climent Casals-Pascual

**Affiliations:** 1 Laboratory of Malaria and Vector Research, National Institute of Allergy and Infectious Diseases, Rockville, MD USA; 2 Hospital Clínic i Provincial de Barcelona, CDB, ISGlobal, Barcelona, Universitat de Barcelona, Barcelona, Spain; 3 Wellcome Trust Centre for Human Genetics, Roosevelt Drive, Oxford, OX3 7BN UK

## Abstract

This opinion article deals with the diagnostic clinical challenges faced by clinicians or health care workers in malaria-endemic areas when a severely sick child presents to the clinic with fever, coma or respiratory distress. Indeed, the coexistence of malaria with other severe infections like meningitis, invasive bacterial infection or pneumonia makes appropriate treatment allocation a matter of life and death. The use of biomarkers has been proposed as a potential solution to this problem. The arrival of high-throughput technologies allowed thousands of molecules (transcripts, proteins and metabolites) to be been screened in clinical samples from large cohorts of well/characterised patients. The major aim of these studies was to identify biomarkers that inform important decisions: should this child be referred to hospital? Should antibiotics, anti-malarials, or both, be administered? There is a large discrepancy between the number of biomarker discovery studies published and the number of biomarkers that have been clinically validated, let alone implemented. This article reflects on the many opportunities and obstacles encountered in biomarker research in malaria-endemic areas.

## Background: the clinical challenge

In malaria-endemic areas, the coexistence of *Plasmodium falciparum* parasitaemia with other severe diseases like pneumonia, bacteraemia and meningitis complicates the definitive diagnosis of severe malaria [1–4]. Notably, when a critically ill child presents with fever, impaired consciousness and *P. falciparum* on the blood smear, severe malaria might not be the primary diagnosis—especially in regions where asymptomatic parasitaemia is common. To further complicate the picture, malaria infection predisposes to bacterial infection and may co-occur with more than half of all cases of bacteraemia in malaria-endemic areas [5]. The presence of malaria parasites or malarial antigens in the child’s blood does not justify withholding empirical parenteral antibiotics [6]. This diagnostic challenge can be further aggravated in settings where medical training or laboratory facilities are inadequate.

The identification and validation of novel biomarkers with high sensitivity and specificity for severe malaria (SM) may contribute to the development and implementation of new diagnostic tools, for example, in a rapid diagnostic test (RDT) format that can optimize treatment allocation and reduce mortality from severe childhood illness.

The current case definition of severe malaria proposed by the World Health Organization (WHO) is based on the observation of parasites on a blood smear accompanied by one or more severe clinical manifestations, namely prostration, impaired consciousness, respiratory distress, multiple convulsions, severe anaemia, jaundice or shock [7]. This definition is pragmatic and rightly prioritizes sensitivity over specificity due to the high risk of death associated with a missed diagnosis or delayed treatment of *P. falciparum* infection. Rapid identification of danger signs by specifically trained non-medical personnel helps to identify those children with malaria at the highest risk of dying. However, despite improved access to treatment and declining trends of malaria observed worldwide [8, 9], the number of deaths remain high (approximately 450,000 yearly [10]), mostly in young children from sub-Saharan Africa.

## What are the limitations of a highly sensitive but poorly specific case definition?

For any clinical condition, the major consequence of a poorly specific case definition is the administration of unnecessary treatment and, in the context of malaria, the potential for accelerated development of parasite drug resistance. According to the WHO recommendation, the administration of anti-malarials in malaria endemic areas should only be used after the presence of parasites has been confirmed through visualization of the parasite *P*. *falciparum* on a Giemsa-stained thick smear (gold standard) or more frequently, through indirect methods, such as the detection of parasite proteins using a RDT. In practice, these guidelines are only partially followed, even when RDTs are available [11].

## Diagnostic specificity is important for appropriate treatment allocation

The diagnostic complexity of severe malaria is a reflection of its pathophysiological complexity, illustrated by three potentially overlapping clinical syndromes of SM: cerebral malaria, respiratory distress and severe malarial anaemia [7, 12]. The majority of deaths from SM occur in children presenting with cerebral malaria or respiratory distress, and those presenting with more than one syndrome of SM have an even greater risk of death. In malaria-endemic regions, severe malaria complicated by impaired consciousness or respiratory distress may be indistinguishable from life-threatening illnesses such as meningitis or pneumonia. In this context, a highly sensitive but poorly specific definition of severe malaria may have deleterious consequences if empirical treatment for alternative diagnoses is not provided.

## How can we improve our ability to discriminate SM from other clinical conditions?

The use of biomarkers has been proposed to facilitate a syndrome-based management approach. In a hypothetical scenario, in a malaria-endemic area a drop of blood could be obtained from a child presenting with fever and respiratory distress and analysed with a panel of biomarkers that have the ability to inform a clinical decision (see Box 1): to administer antibiotics (pneumonia), anti-malarials (*P. falciparum* or non-falciparum malaria), administration of antibiotics and anti-malarials, to indicate urgent referral to hospital [13–15], or send the child home with symptomatic treatment or no treatment at all (non-severe respiratory virus, or common cold). To achieve this, the biomarker panel should determine the presence or absence of the common critical illness in the community, namely severe malaria, bacterial infection, or viral infection.

Which biomarkers should be used? And do these biomarkers exist? The quest to identify the perfect panel of biomarkers to inform clinical decisions was galvanized by the advent of high-throughput “omics” technologies (see Box 2). When these powerful technologies became more popular and affordable, clinical samples from well-characterized cohorts of patients were screened for potential biomarkers. Exhaustive lists of genes, mRNA and proteins were produced and candidate markers identified. Unfortunately, difficulties in candidate selection and the need for thorough clinical validation mean that very few putative biomarkers have been clinically implemented.

## Machine-learning or machine-teaching?

Candidate selection is problematic. The identification of a minimal set of informative biomarkers relies heavily on machine-learning algorithms. Briefly, an algorithm is given two phenotypes, i.e. mild malaria versus severe malaria, and it identifies discriminating features from a broad dataset (e.g., genes, transcripts, proteins or metabolites). Some of these features are then selected and tested in silico on a different set of patients not used in the training phase. In a successful test, the algorithm will correctly assign the unknown patients to the correct clinical phenotype based on a minimal set of informative features. The list of features can vary in length depending on the type of biomarker and the study design. In proteomic and transcriptomic studies, it is not uncommon to identify hundreds of discriminant features. The more distinct the clinical phenotypes analysed, the longer the list of discriminating biomarkers. It is important that the study design mirror the diagnostic challenge to be resolved. A study design that includes only extreme phenotypes (for example, severe malaria *versus* healthy controls) may not yield relevant information and the candidate biomarker(s) might just be expensive alternatives to measuring axillary temperature. Instead, a more useful approach might be to compare severe malaria *versus* mild malaria, or severe malaria versus pneumonia, to identify biomarkers that inform clinical decision-making.

## How much evidence is sufficient to validate candidate biomarkers?

Biomarker validation is the most expensive, risky and time-consuming phase of biomarker research and rarely pursued by investigators unless the results of the discovery phase are very promising. Here, the investigators are expected to accurately quantify the candidate biomarkers in biofluids and produce estimates of sensitivity, specificity, positive- and negative-predictive values to inform the research community about the potential clinical impact of the candidate biomarker(s). Next, independent, adequately powered, clinical studies will be required, followed by at least one larger, ambitious prospective study where clinical decisions are based on the biomarker concentration and patient outcomes are compared to those achieved with standard practice. This phase also depends on the existence of suitable intellectual property and the availability of a diagnostic product or a prototype to measure the biomarker. In reality, few examples of successful biomarkers have successfully emerged from this process. Even when efficacy is demonstrated, cost-efficacy may prevent incorporation of a biomarker into routine practice. Procalcitonin (PCT), a biomarker that detects bacterial infection, can be used to illustrate this point (reviewed in [16]). A large number of adequately powered studies have shown the high efficacy of PCT to identify bacterial infection, including a clinical trial conducted to ascertain the value of this molecule to successfully indicate antibiotic treatment in critical care patients [17]. However, when it comes to clinical use, the cost of measuring PCT could be 10–20 times higher than a less biomarkers such as C-reactive protein. How do you determine when the increased performance of a biomarker justifies the increased cost? Attempts at cost-effectiveness modelling have not resolved this issue. In the case of malaria, the cost of an optimal panel of biomarkers will have to be affordable, and certainly not cost more than the treatments it saves, if it is to be implemented in practice.

## A closer look at cerebral malaria

Not all good biomarkers are molecules. A working definition of “biomarker” (short for “biological marker”) refers to objective indications of a medical state observed from outside the patient [18]. Although the term “biomarker” has evolved to become equivalent to molecular marker, a clinical sign can be also labelled as biomarker if it can be quantified accurately and reproducibly.

Cerebral malaria (CM) provides a good example. CM is classically defined in children as a deep level of unconsciousness (inability to localize a painful stimulus) in the presence of asexual parasitaemia, after the correction of hypoglycaemia and exclusion of other encephalopathies, especially bacterial meningitis and locally prevalent viral encephalitis [19]. Notably, this definition underlines diagnostic specificity by emphasizing the need to rule out other conditions. It is not always trivial to discriminate cerebral malaria from meningitis as both conditions share many clinical features [3, 20]. Moreover, post-mortem studies have shown that even when strict clinical criteria are used to diagnose CM, 23% of cases were found on post-mortem examination to have died from other causes [21–23]. Interestingly, this and other studies describe specific retinal changes as the only clinical sign that discriminates malarial from non-malarial causes of coma [22, 23]. While there are logistical impediments to routine implementation of fundoscopy in hospitals and clinics, this specific clinical sign offers an opportunity to better define cerebral malaria for molecular biomarker discovery studies. Indeed, angiopoietin-2 has been proposed as a candidate biomarker to discriminate CM from other causes of coma as this biomarker appears to be closely associated with the retinal changes of CM in Malawian children [24].

Molecular biomarkers that discriminate SM syndromes from other infections are urgently needed. Although some of the biomarker research show promise (reviewed in [25]), a major bottleneck to implementation remains the development of effective, rapid, affordable, easy-to-use diagnostic tools that should ideally combine pathogen and host-derived biomarkers. Syndrome-based diagnostics may well replace pathogen-based diagnostics in the future. Academy-industry partnerships will be necessary to shepherd promising biomarkers from discovery through implementation.

## Box 1. Severe malaria versus bacterial pneumonia. Some progress

Respiratory distress is a common presentation of SM, but practically impossible to distinguish from severe pneumonia [4, 26–28]. Critically, the clinical management is different for both conditions. It is, therefore, crucial to develop new tools to assist health workers to effectively identify and refer to hospital those children with malaria or pneumonia who are at the highest risk of dying.

In 2014, Huang and colleagues conducted a proteomic discovery study in 390 Gambian children and identified a panel of two biomarkers (**haptoglobin and lipocalin-2**) to discriminate acute respiratory infection from severe malaria with respiratory distress [29]. These proteins were further validated in a study of 293 Kenyan children. The combination of haptoglobin and lipocalin-2 discriminated acute respiratory distress from malaria with high sensitivity and specificity (area under the ROC curve of 99% in Gambian children and 82% in Kenyan children). More recently, a study conducted in Mozambique [30], used a multiplex immunoassay panel of 56 proteins, to find that the combination of three proteins (**haptoglobin, tumour necrosis factor receptor 2 or IL-10, and tissue inhibitor of metalloproteinases-1**) discriminated bacterial diagnosis from malaria with high sensitivity and specificity (96 and 86%, respectively).

*Why is haptoglobin a good biomarker to discriminate malaria from bacterial infection in children with respiratory distress*?

Haptoglobin is a highly abundant plasma protein. During acute inflammatory response, haptoglobin is further secreted by neutrophils in response to infection [31]. However, the primary function of haptoglobin is to bind free-circulating haemoglobin and prevent haemoglobin-induced oxidative cell damage. These high-affinity complexes are later removed by CD163 scavenger receptor on macrophages that will eliminate this complex (and recycle iron) through endocytosis and subsequent intracellular degradation [32]. Acute malaria infection is accompanied by some degree of haemolysis, and the drive to increase haptoglobin as part of the inflammatory response is clearly offset by haemolysis, which drives haptoglobin concentration towards depletion. This polarized response (increased in bacterial infection and decreased in malaria) produces a much sought after “curtain effect” that pulls the two conditions apart by quantifying a single biomarker.

## Box 2. Challenges and opportunities of clinical proteomics in biomarker discovery

Peptides and proteins define the actual phenotype of an organism and thus reflect physiological and pathophysiological processes. The identification of peptide/proteins that are associated with particular phenotypes may be used for diagnostic/therapeutic purposes. One of the biggest challenges for large-scale proteomic studies is to identify peptides/proteins across a wide dynamic range (10 orders of magnitude). In this context, the combination of (1) depletion of the most abundant proteins, (2) pre-analytical sample fractionation and (3) protein digestion and MS/MS-based peptide sequencing (shotgun proteomics) can identify peptides at concentrations of ng/mL, which falls three orders of magnitude short of a complete proteome analysis (pg/mL) [33]. However, this limitation does not preclude the use of these techniques to identify clinically useful markers. Moreover, advances in mass spectrometry lead towards high-throughput analysis with improved chromatographic stability, allowing for fast proteomic profiling with quantitative information for hundreds of proteins in individual patients.
